# Comparative genomic analysis of a naturally competent *Elizabethkingia anophelis* isolated from an eye infection

**DOI:** 10.1038/s41598-018-26874-8

**Published:** 2018-05-31

**Authors:** Eswarappa Pradeep Bulagonda, Bhavani Manivannan, Niranjana Mahalingam, Manmath Lama, Pachi Pulusu Chanakya, Balaram Khamari, Sudhir Jadhao, Madavan Vasudevan, Valakunja Nagaraja

**Affiliations:** 1 Department of Biosciences, Sri Sathya Sai Institute of Higher Learning, Prasanthi Nilayam, Puttaparthi, Andhra Pradesh India; 2 Department of Microbiology, Sri Sathya Sai Institute of Higher Medical Sciences, Prasanthigram, Andhra Pradesh India; 3 Bionivid technology Pvt Ltd, 4C-209 4th cross, Kasturi Nagar, Bengaluru, India; 4 Department of Microbiology and Cell Biology, Indian Institute of Science, Bengaluru, India; 5 Jawaharlal Nehru Centre for Advanced Scientific Research, Bengaluru, India

**Keywords:** Clinical microbiology, Bacterial genetics

## Abstract

*Elizabethkingia anophelis* has now emerged as an opportunistic human pathogen. However, its mechanisms of transmission remain unexplained. Comparative genomic (CG) analysis of *E. anopheles* endophthalmitis strain surprisingly found from an eye infection patient with twenty-five other *E. anophelis* genomes revealed its potential to participate in horizontal gene transfer. CG analysis revealed that the study isolate has an open pan genome and has undergone extensive gene rearrangements. We demonstrate that the strain is naturally competent, hitherto not reported in any members of *Elizabethkingia*. Presence of competence related genes, mobile genetic elements, Type IV, VI secretory systems and a unique virulence factor arylsulfatase suggests a different lineage of the strain. Deciphering the genome of *E. anophelis* having a reservoir of antibiotic resistance genes and virulence factors associated with diverse human infections may open up avenues to deal with the myriad of its human infections and devise strategies to combat the pathogen.

## Introduction

*Elizabethkingia* belonging to the family *Flavobacteriaceae* is a genus with 4 known species: *E. meningoseptica, E. anophelis, E. miricola and E. endophytica*. Since its discovery in 1959, *E. meningoseptica*^1^ has been described to be involved in diverse human infections such as meningitis, keratitis and sepsis among immunocompromised individuals^2,3^. In 2003, *E. miricola* was reported for the first time from the condensed water samples of the Russian space station Mir^4^. *E. miricola* was later found to be associated with sepsis, bacteraemia and pneumonia^5^. *E. endophytica* isolated from corn has not yet been associated with any of the human infections^6^. In 2011, *E. anophelis* was discovered from the midgut of *Anophelis gambiae* mosquito^7^. It has been linked to neonatal meningitis^8^, nosocomial outbreaks^9^ and catheter associated infections with high mortality rates^10^.

*Elizabethkingia* strains have been found to encode metallo-betalactamases conferring carbapenem resistance^11^. Because of the bacterium’s innate resistance to several classes of antibiotics, treatment of *Elizabethkingia* infections is challenging^3^. Several studies have reported that patients with severe underlying diseases and a history of antibiotic exposure are more susceptible to *Elizabethkingia* related nosocomial infections^2^. Scant information is available on the potential modes of infection and their ability to adapt to diverse host environments. Hence, analysis of their genomes to study the potential for horizontal gene transfer leading to enhanced infection capabilities and survival abilities across various ecological niches assumes additional significance.

Here, we have investigated a new *E. anophelis* endophthalmitis strain, a multidrug resistant pathogen isolated from a post-operative endophthalmitis patient^12^. After sequencing, CG analysis was performed with twenty-five other *E. anophelis* strains whose genomes were publicly available to get an insight into their phylogenetic position and also to understand the similarities and differences in their gene contents with the aim of determining the unique features of the organism isolated for the first time from an eye infection. Although the genome of the organism has overall similarities with the genomes of other *E*. *anophelis* strains, presence of several unique genes and the ability of horizontal gene transfer indicate its distinct origin.

## Results

### Characteristics of the patient

The patient was diagnosed with post-operative endophthalmitis (anterior chamber hypopyon, dense vitreous haze with yellow reflex). There was no view of the retina and his vision was only perception of light. Subsequently, vitrectomy was performed to debulk the infection and pus was removed from the infected eye. Empirical treatment for endophthalmitis was immediately initiated.

### Identification and antibiotic susceptibility profile

The isolate was initially identified as *Elizabethkingia meningoseptica* by Vitek-2. Subsequently, with the availability of additional *Elizabethkingia* genomes and 16 s rDNA sequence analysis, the genome of the study isolate was re-identified as *Elizabethkingia anophelis*. MIC analyses of 13 antibiotics revealed that the pathogen was resistant to penicillins, cephalosporins, monobactam, carbapenems, aminoglycosides and trimethoprim/sulfamethoxazole; and sensitive to levofloxacin and minocycline. The organism exhibited intermediate resistance to tigecycline and ciprofloxacin (Table 1).

**Table 1:** Antibiotic susceptibility profile of *E. anophelis* endophthalmitis.

S.no	Antibiotic Class	Antibiotic	MIC (µg/ml)	Resistance profile
1	Penicillins	Piperacillin/tazobactam	>=128	Resistant
2	Cephalosporins	Ceftazidime	>=64	Resistant
3		Cefepime	>=64	Resistant
4	Monobactum	Aztreonam	>=64	Resistant
5	Carbapenems	Imipenem	>=16	Resistant
6		Meropenem	>=16	Resistant
7	Aminoglycosides	Amikacin	>=64	Resistant
8		Gentamicin	>=16	Resistant
9	Quinolones	Ciprofloxacin	2	Intermediate Resistant
10		Levofloxacin	2	Sensitive
11	Tetracyclines	Minocyclin	<=1	Sensitive
12		Tigecycline	4	Intermediate Resistant
13	Trimethoprim	Trimethoprim/Sulfamethoxazole	80	Resistant

Antibiotic susceptibility test was performed using Vitek-2 as per clinical laboratory standards Institute (CLSI) guidelines. MIC – minimum inhibitory concentration of the antibiotic in µg/ml.

### Comparative analysis of core and pan genome

**Figure 1:**
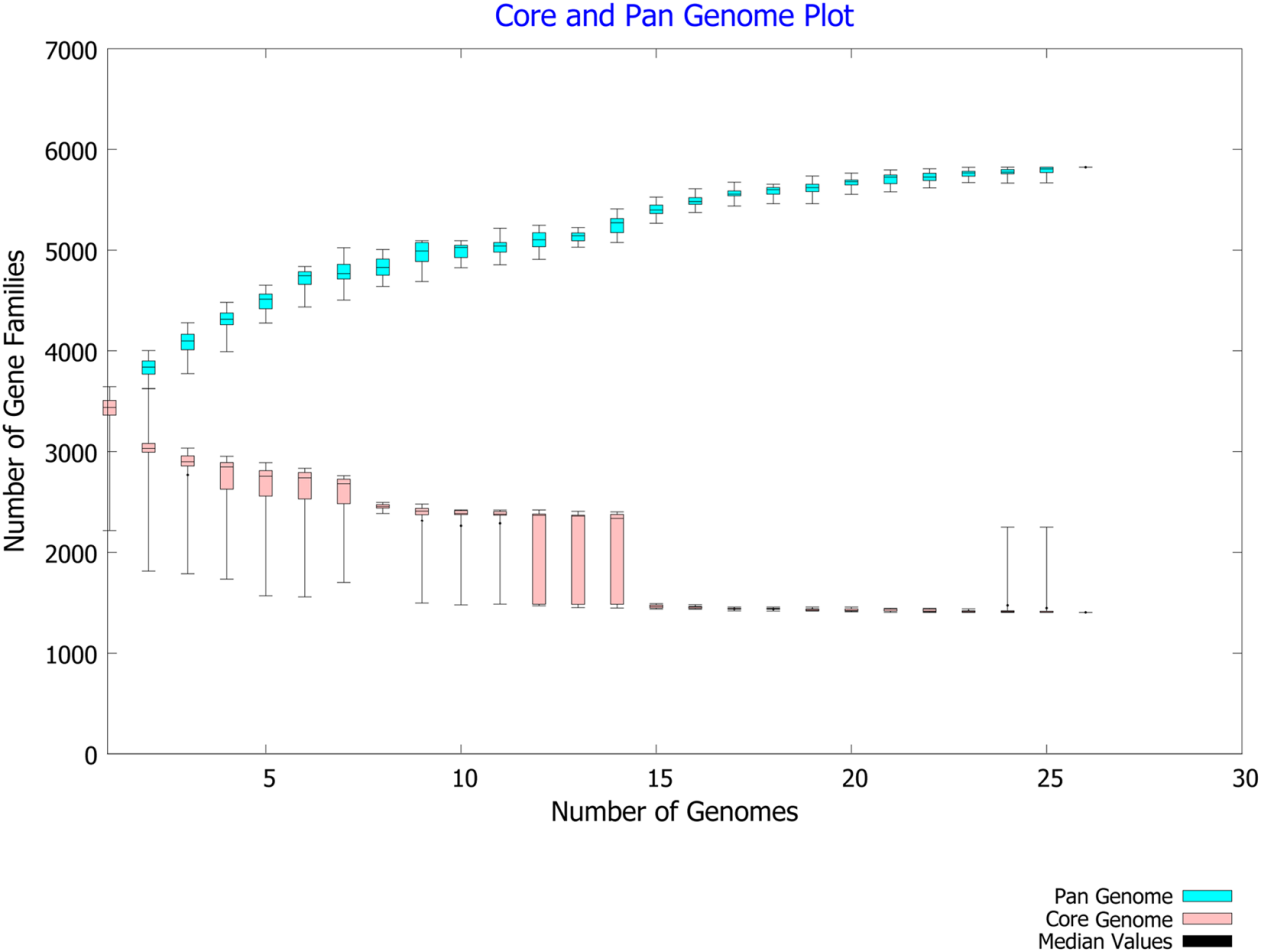
The Core and pan genome of the compared 26 *E. anophelis* genomes. A total of 26 *E. anophelis* genomes have been analysed using the default parameters of the BPGA pipeline. The analysed *E. anophelis* genomes share 1404 core genes. Cyan colored boxplots indicates the change in number of pan-genome gene groups to the number of genomes added sequentially. Pink boxplots indicate the change in number of core-genome gene groups to the number of genomes added sequentially. A positive correlation is observed between the pan genome orthologous groups (POGs) and the genomes under analysis in the pan genome curve, while there is a negative correlation in the core-genome curve as the number of POGs were observed to decrease with the increase in the number of genomes.

**Figure 2:**
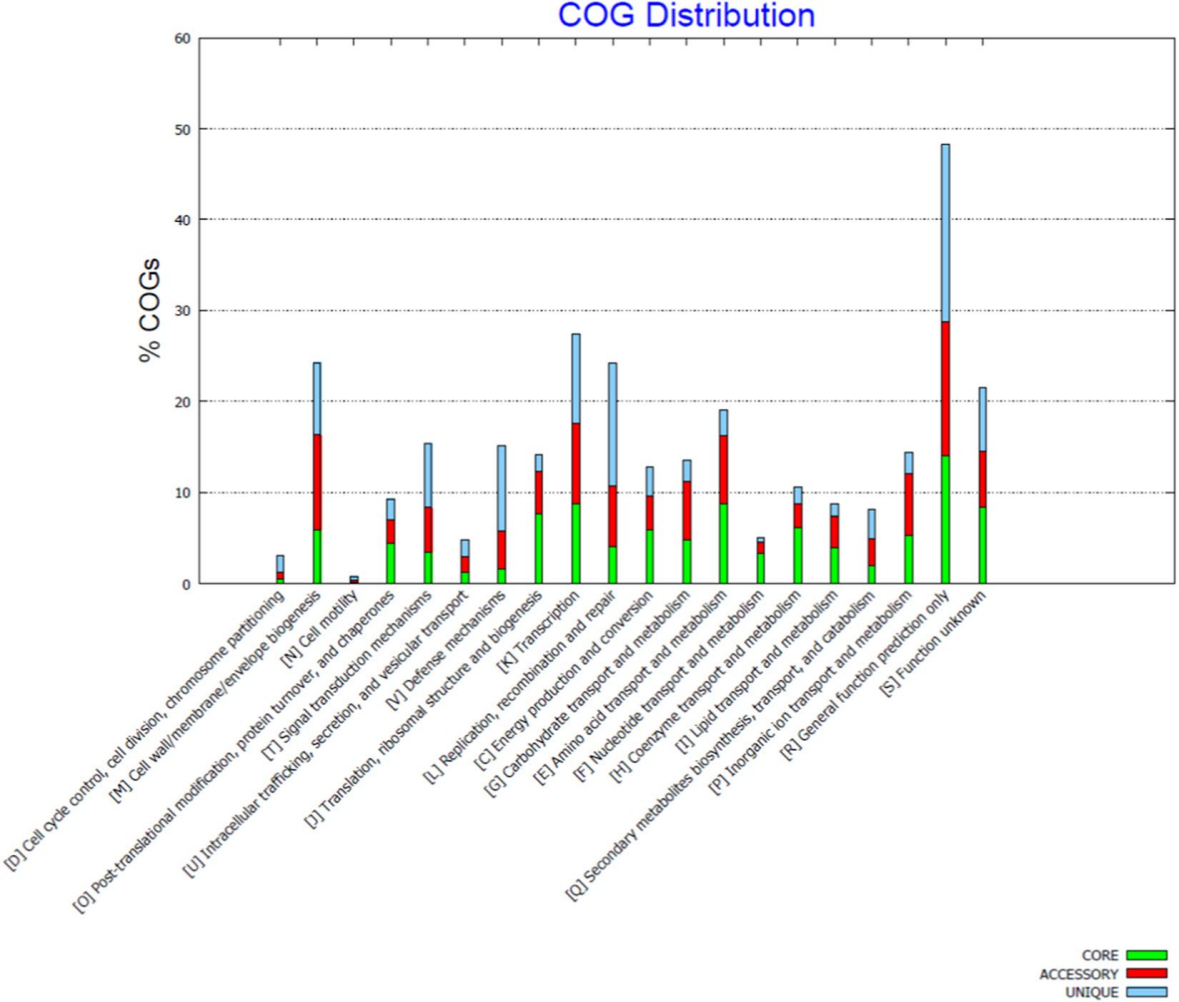
Cluster of Orthologous Groups (COG) analysis of the 26 *E. anophelis* genomes. Comparison of the COG distribution between the core, accessory and unique genes of the 26 *E. anophelis* strains has been analysed using the default parameters of the BPGA pipeline. The COG categories are presented on the X-axis and the percentage of the genes enriched in each category of the COG classes are indicated on the Y-axis.

**Figure 3:**
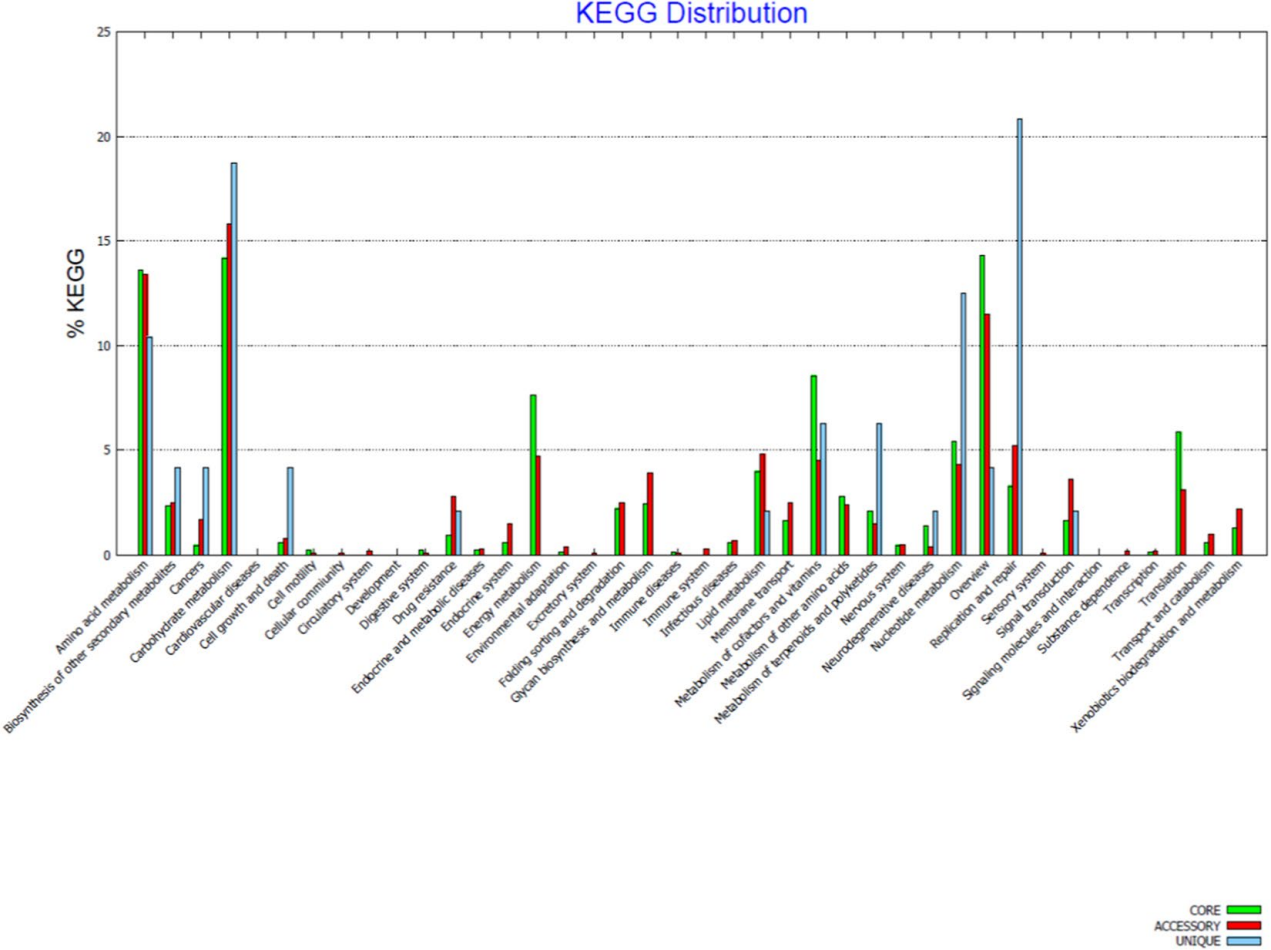
KEGG analysis of the 26 *E. anophelis* genomes. Functional annotation of the core, accessory and unique genes of the 26 *E. anophelis* genomes has been performed using the default settings of the BPGA pipeline. The KEGG categories are mentioned on the X-axis and the percentage of the genes associated with each of the KEGG category are presented on the Y-axis.

26 genomes of *E. anophelis* including the genome of the new *E. anophelis endophthalmitis* study strain were retrieved from NCBI database for CG analysis. The average size of the genomes is 4.03 Mb and average G + C% is 35.61%. The strain with the maximum size is NUHP1 (4.36 Mb) and the smallest genome is As1 (3.59 Mb) (Table 2). The predicted protein sequences of all the 26 *E. anophelis* genomes were used as input to conduct the core-pan genome analysis. CG analysis revealed that 1404 (40.79%) genes were shared between all the 26 strains, which may be considered to be “core genome”. The accessory genome varied from 656 to 2240 genes (avg. 59.2%) across the strains (Fig. 1). Most notably, exclusion of the study isolate’s genome from the analysis led to an inclusion of an additional 844 genes in the core genome of the remaining 25 genomes. The study strain was found to have maximum number of exclusively absent (846) and unique (156) genes (Table 3), revealing that it has an open pan genome. Annotation of the pan-genome of all the 26 genomes to understand the enrichment of the COG (Fig. 2) and KEGG pathways (Fig. 3) has been mapped.

**Table 2:** Comparison of genome characteristics of the 26 *E. anophelis* isolates used in analysis.

Strain Name	Year of Collection	Accession id	Genome size in Mb	GC %	No. of Contigs	Genes	Proteins
0422	1950	NZ_LNOG01000011	3.9599	35.6	26	3634	3548
502	2012	NZ_AVCQ01000001	3.96066	35.5	21	3617	3520
12012-2PRCM	2009	NZ_LPXG01000011	4.02331	35.6	83	3671	3554
Ag1	2010	NZ_AHHG01000001	4.04571	35.5	51	3723	3572
As1	2013	NZ_LFKT01000006	3.59087	35.5	12	3303	3229
B2D	2013	NZ_JNCG01000007	3.93625	35.5	50	3553	3473
CSID_3000521207	2016	NZ_CP015067	3.85345	35.7	1	3490	3390
CSID_3015183678	2016	NZ_CP014805	3.93122	35.8	1	3562	3461
CSID_3015183681	2016	NZ_CP015068	3.93122	35.8	1	3563	3461
CSID_3015183684	2016	NZ_CP015066	3.93122	35.8	4	3562	3458
EM361-97	2010	NZ_KV757122	4.08405	35.7	27	3729	3614
Endophthalmitis	2014	JSAA01000100	4.01982	35.5	167	3729	2302
FMS-007	Not available	NZ_CP006576	3.93897	35.6	1	3578	3470
LDVH-AR107	2004	NZ_FTPG01000001	3.98893	35.7	108	3658	3538
NUH1	2012	NZ_ASYH01000001	4.33466	35.6	59	3993	3879
NUH11	2012	NZ_ASYK01000005	4.09148	35.6	59	3757	3642
NUH4	2012	NZ_ASYI01000001	4.23949	35.6	50	3912	3811
NUH6	2012	NZ_ASYJ01000011	4.1238	35.6	74	3812	3696
NUHP1	2012	NZ_CP007547	4.36983	35.6	1	4016	3898
NUHP2	2012	NZ_ASYF01000003	4.33465	35.5	59	3988	3882
NUHP3	2012	NZ_ASYG01000009	4.33411	35.5	71	3985	3871
Po0527107	2006	NZ_CCAC010000089	4.03206	35.5	89	3674	3573
PW2806	2012	NZ_CBYD010000038	3.91281	35.9	388	3612	3456
PW2809	2012	NZ_CBYE010000026	3.92215	35.8	278	3598	3452
R26	2006	NZ_ANIW01000066	4.03272	35.4	66	3726	3633
V0378064	2011	NZ_CCAB010000170	4.03675	35.7	214	3648	3556

**Table 3:** Core, accessory, unique and exclusively absent genes in the 26 *E. anophelis* genomes after pan-core genome analysis using BPGA pipeline.

Strain Name	No. of core genes	No. of accessory genes	No. of unique genes	No. of exclusively absent genes
0422	1404	1950	94	3
502	1404	1888	108	2
12012-2PRCM	1404	1930	131	10
Ag1	1404	2010	10	30
As1	1404	1693	3	149
B2D	1404	1868	101	5
CSID207	1404	1896	0	27
CSID678	1404	1958	0	0
CSID681	1404	1959	0	1
CSID684	1404	1956	0	0
EM361-97	1404	1964	113	3
Endophthalmitis	1404	656	156	846
FMS007	1404	1913	56	6
LDVH-AR107	1404	2006	21	16
NUH1	1404	2240	0	0
NUH4	1404	2185	22	0
NUH6	1404	2107	33	5
NUH11	1404	2097	12	1
NUHP1	1404	2236	0	0
NUHP2	1404	2236	0	0
NUHP3	1404	2238	2	0
Po0527107	1404	2032	45	0
PW2806	1404	1956	3	12
PW2809	1404	1948	5	13
R26	1404	2052	38	0
V0378064	1404	2035	20	0

Core genes – number (No.) of genes that are shared by all the study genomes, Accessory genes – Genes that are not shared by all the genomes, Unique genes – genes that are found exclusively in a particular genome, exclusively absent genes – genes that are exclusively absent in a particular genome but are otherwise found in the other genomes.

### Core genome based phylogenetic analysis

**Figure 4:**
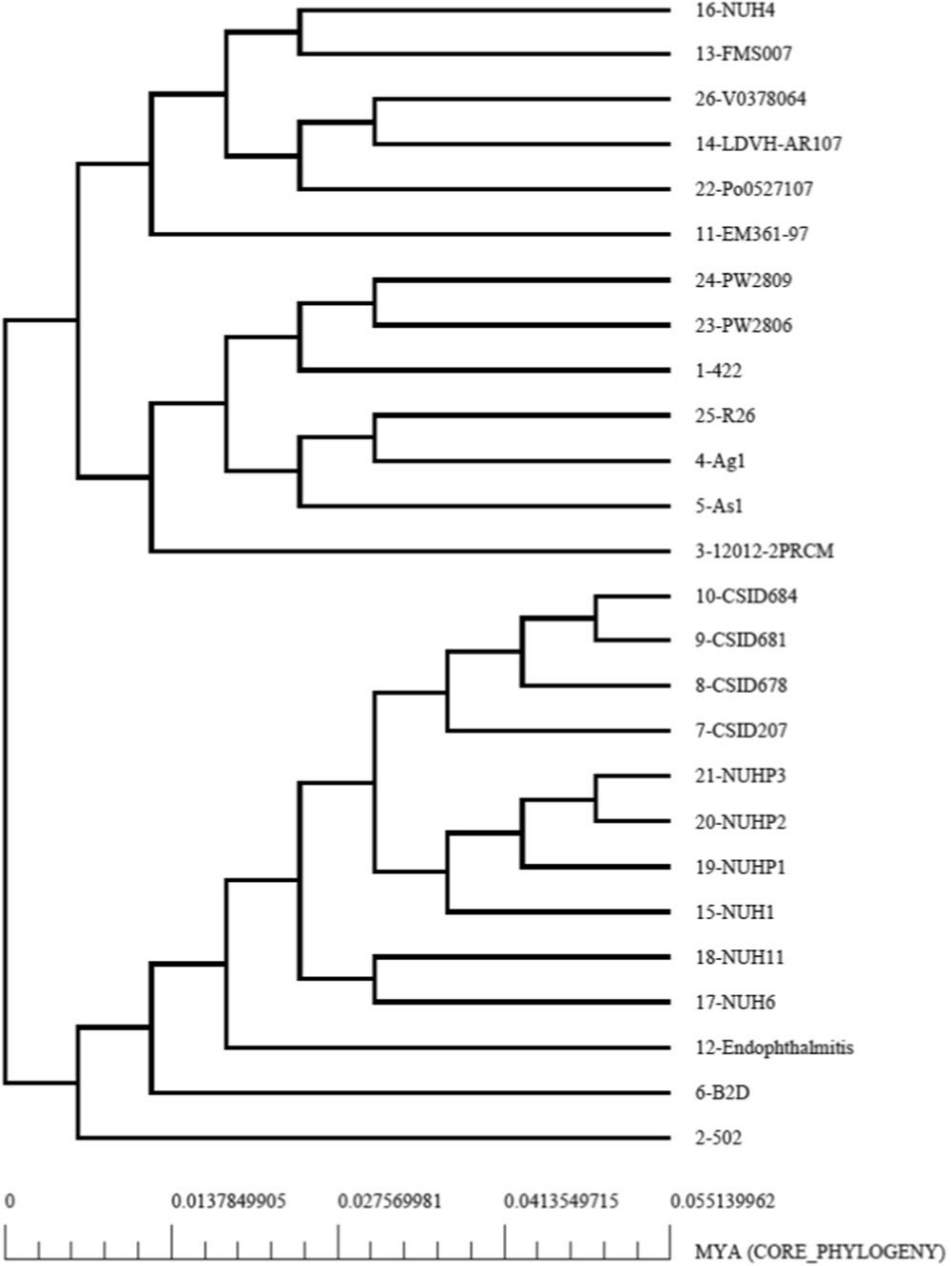
Core Phylogenetic tree: The core phylogenetic tree is based on the protein sequences of the concatenated core genes (1404) of the 26 *E. anophelis* genomes. The tree was generated by Neighbor Joining Method in MUSCLE using default parameters. The tree was plotted using the Perl package Bio::Phylo::Treedrawer available at http://search.cpan.org/~rvosa/Bio-Phylo-v2.0.1/lib/Bio/Phylo/Treedrawer.pm as implemented in BPGA pipeline. The scale bar presented at the foot of the tree indicates time period in millions of years (MYA).

Phylogenetic tree was constructed to infer the relationship among these genomes using the concatenated sequences of 1,404 core proteins identified from the pan/core genome analysis. The tree separated the 26 *E. anophelis* genomes into two distinct branches. In the lower branch, genomes of 502, B2D, Endophthalmitis were found to be closely related. Genomes of *E. anophelis* from Singapore have clustered together (NUH6, NUH11, NUH1, NUHP1, NUHP2, NUHP3) excepting NUH4. Further, the genomes of the four wisconsin outbreak isolates CSID300521207, CSID3015183678, CSID3015183681 and CSID3015183684 have been found to cluster together^13^. The other branch was found to have two sub clusters. 12012-2PRCM, As1, Ag1, R26, 0422, PW2806 and PW2809 genomes grouped into one, while EM361-97, Po0527107, LDVH-AR107, V0378064, FMS007 and NUH4 genomes were part of the second sub cluster (Fig. 4). The genome of the isolated strain included in the lower branch, is phylogenetically closer to the strains B2D and 502. However, unlike the test isolate being associated with endophthalmitis, the strains B2D and 502 were isolated from dental plaque and traumatic wound respectively.

### Resistance to antibiotics and toxic compounds

Screening of all the study genomes revealed the presence of *bla*GOB, *blab* and *bla*CME (excepting strain AS1) beta-lactamase genes in most of the strains. Further, vancomycin (VanW) resistance was predicted among all the 26 strains. A gene encoding bile salt hydrolase - Choloylglycine hydrolase (EC 3.5.1.24) was found in all the analysed genomes excepting As1. This enzyme has been previously reported to protect *Brucella abortus* in the host gut from the toxic and antimicrobial activity of the bile salts^14^. Genes associated with CzcCBA, a membrane bound protein complex aiding in heavy metal resistance^15^ have been identified in all the 26 genomes. Genes coding for proteins conferring resistance to several heavy metals (copper, Zinc, cadmium, cobalt) have been discovered among all the study isolates. In addition, genes coding for the proteins leading to arsenic resistance have been predicted in all the genomes. Further, some of the strains (NUH1, NUH11, NUH4, NUH6, NUHP1, NUHP2 and NUHP3) were found to possess arsenic resistance operon repressor suggesting the inducibility of the system’s resistance. Several genes encoding for the multidrug efflux pumps including RND (CmeB – 26 strains, CmeC – 14 strains), multi antimicrobial extrusion protein (Na(+)/drug antiporter – 26 strains) belonging to the MATE family of MDR efflux pumps and acriflavine resistance protein (RND efflux pump transporter – 26 strains)^16^ were found in the study genomes (Supplementary Table 2).

### Putative virulence and anti-virulence genes

Many genes that may be associated with invasion and intracellular resistance in humans have been identified. We have identified homologs of the gene encoding for an agmatine deiminase in all the 26 isolates. Agmatine deiminase has been reported to aid growth at low pH and biofilm formation, confer acid tolerance in addition to being a potential adherence factor in the colonization of vagina^17^. Putative hemolysin and a hemolysin secretion protein have been predicted among all the 26 genomes. Hemolysin has been implicated as a virulence factor among several gram-negative and gram-positive pathogens^18^. It has also been reported that hemolysin could be a potential ocular virulence factor in *Bacillus cereus* and *Staphylococcus aureus* leading to endophthalmitis^19,20^ and keratitis^21^ respectively. However, among the 26 genomes included in this study, arylsulfatase has been identified only in the current study isolate. Arylsulfatase has been implicated previously in *E. coli* infection of the brain microvascular endothelial cells (BMEC) of the host. Presence of arylsulfatase may contribute to the ability of the pathogen to cross the blood-brain barrier leading to meningitis^17,22^. An operon consisting of Quinolinate synthetase [EC 2.5.1.72], L-aspartate oxidase [EC 1.4.3.16], and quinolinate phosphoribosyl transferase [EC 2.3.2.19] involved in quinolinate biosynthesis has been identified in all the genomes being analysed (Supplementary Table 3). These genes are considered to have potential anti-virulence function as their activation was reported to inhibit invasion and intracellular spread among *Shigella* species^23^.

### Analysis of Mobile Genetic Elements (MGEs)

MGEs are the major contributors for Horizontal Gene Transfer (HGT). A total of 59 prophage related regions have been identified across the study genomes. Of these, 56 of them appear to be incomplete prophage genomes in 23 study isolates. The other three strains (422, PW2806 and PW2809) were predicted to contain questionable prophage genomes. The size of the prophage genomes varied from 5.6 kb to 37.4 kb. Strains NUH1, NUH4, NUHP1, NUHP2 and NUHP3 were found to harbor maximum number of prophage regions (4 numbers) compared to the others. The average G + C% among these prophage genomes was 34.55% compared to the average G + C% of the study genomes (35.62%) (Supplementary Table 4).

A total of 107 Genomic Islands (GIs) have been identified in 25 of the study genomes excepting B2D. The smallest of the predicted GI was 8.2 kb in the strain EM361-97 and the largest GI was 31.8 kb in NUH1 and NUHP3 strains. NUH1, NUH4 and NUHP2 were found to harbor 8 GIs. Maximum number of coding DNAs (66 numbers) were found in the GI (region III) of the strain 422. Several virulence factors, antibiotic resistance genes, pathogenicity islands, insertion sequences, prophage related genes and genes of the secretion systems have been identified in these GIs (Supplementary Table 5). Further, remnants of several Integrative and Conjugative Elements (ICEs) have been found in many of the study genomes.

### Type VI and Type IV Secretion systems

T6SS plays an important role in bacterial pathogenesis by allowing the transport of virulence factors, targeting the host cells as well as helping in competing with other bacteria in their niche^24^. They are widely distributed in the genomes of the phylum Bacterioidetes of which Flavobacteriaceae is a family. More specifically, T6SS^iii^ has been reported to be prevalent among the members of the Flavobacteriaceae^25^. Consistent with earlier reports^25,26^, several of the study genomes were found to possess T6SS^iii^. Genes annotated to TssN, TssO and TssP proteins which are unique to only T6SS^iii^ along with those coding for the other core components such as TssB, TssC and extracellular components VgrG and HcP have been identified in twenty-four genomes. Only strains NUH6 and As1 did not appear to harbor any genes coding for the T6SS components (Supplementary Table 6).

T4SS are established components of bacterial conjugation and virulence. T4SS genes are also acquired as part of the Integrative and Conjugative Elements (ICEs). Our analysis revealed that 23 of the genomes possessed the genes associated with T4SS but are absent in strains As1, B2D and R26 (Supplementary Table 7).

### Defence and repair systems

Bacteria employ a host of mechanisms to protect themselves from the invading genetic elements. These include Restriction Modification systems (RMs) which are considered to be innate immune systems and Clustered Regularly Interspaced Short Palindromic repeat sequences (CRISPRs), considered to be adaptive immune systems^27^. Our analysis revealed the presence of Type I and Type II RM systems. Unlike all other genomes analyzed in the study, *E. anophelis* endophthalmitis was found to possess maximum number of RM system associated genes. The genome was found to possess 12 RM genes, while the strains 502 and B2D did not possess any genes coding for RM systems (Supplementary Table 8). Analysis for CRISPRs indicated that only four of the study genomes (FMS007, LDVH-AR107, Po0527107 and V0378064) possess confirmed CRISPRs (Supplementary Table 9). Analysis for the presence of anti-restriction systems^28^ led to the detection of an anti-restriction gene ArdA among 19 of the study genomes. ArdA protein was reported to support the MGEs in evading the Type I RM systems and augment the spread of resistance determinants^29^ (Supplementary Table 10).

CG analysis indicated that most of the DNA repair pathways are represented in all the study genomes. Most of the repair pathways appear to be intact. In the majority of the genomes excepting those of the wisconsin strains, there was no disruption in the *mutY* (Adenine DNA glycosylase) gene. In the genomes of the four Wisconsin strains, the 1,029 bp *mutY* gene was found to be disrupted by the insertion of a 62,212 bp ICE*Ea1* (Supplementary Fig. 1)^13^. A total of 32 protein coding genes (excluding hypothetical genes) involved in transposition, excision of the conjugative transposon, heavy metal resistance and tetracycline resistance have been found inside the ICE.

### *E. anophelis* endophthalmitis is naturally competent

Although a number of *Elizabethkingia* strains have been identified and characterized, natural transformation has not yet been reported in any of them. Given the presence of a considerable number of GIs and other gene clusters possibly acquired through HGT in the strain, we investigated the capability of the organism to carry out HGT. In the absence of any well characterized bacteriophages for these group of bacteria, we resorted to study natural transformation.

**Figure 5:**
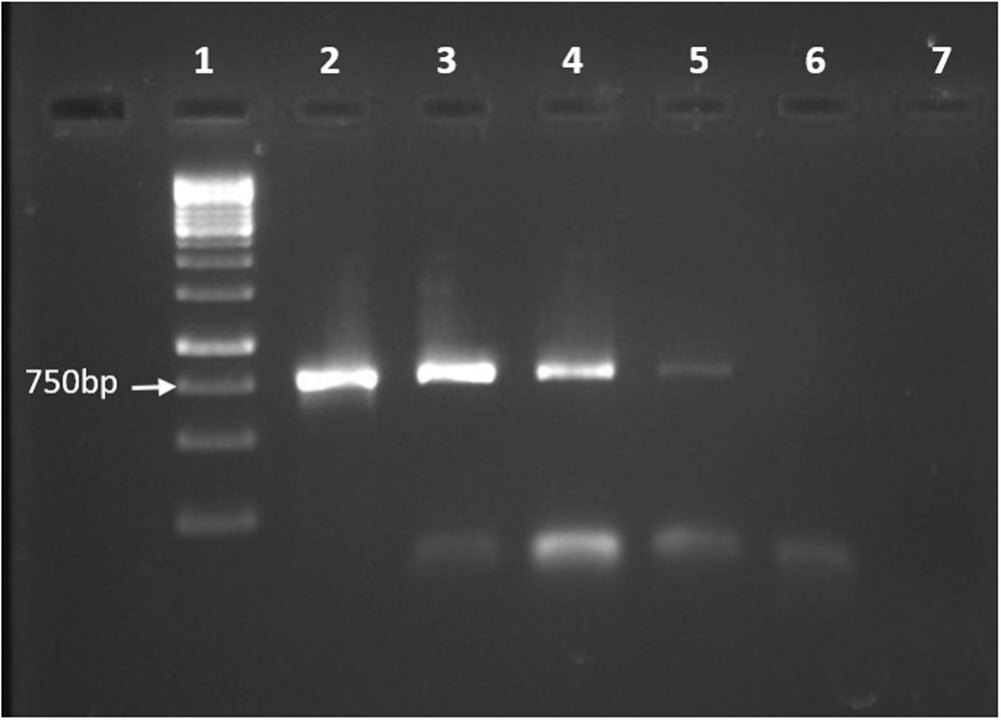
PCR confirming the natural transformation in *E. anophelis* endophthalmitis. Lane 1: 1 kb DNA ladder. Lane 2: Positive control (pCMV-6 BDNF plasmid used as template). Expected band size of BDNF gene is 750 bp. Lane 3–5: PCR products from plasmid isolated from *E. anophelis* endophthalmitis after natural transformation. Lane 6–7: Negative controls.

Natural transformation was observed after exposing plasmid DNA to exponentially growing cells. PCR analysis for the presence of BDNF gene in the plasmids isolated from the transformants confirmed that *E. anophelis* is naturally competent (Fig. 5). However, natural competence was observed only when OD_600_ reached 0.84. Genome analysis revealed three genes – (a) DNA internalization-related competence protein ComEC/Rec2, (b) Competence protein F homolog and (c) Competence/damage-inducible protein CinA involved in DNA internalization and transformation. These genes are present in majority of the analyzed genomes indicating that natural transformation may be occurring in other *Elizabethkingia* (Supplementary Table 11).

## Discussion

In this manuscript, we have described the uncommon features of a new strain of *E. anopheles* isolated from a post-operative endophthalmitis patient. This is the first ever isolate of the species from a patient suffering from this infection. Initial Vitek-2 analysis from the vitreous fluid of the patient led to the identification of the organism as *Elizabethkingia meningoseptica*^2^. Availability of several *Elizabethkingia* genomes due to the advent of whole genome sequencing and 16srDNA analysis lead to the unambiguous identification of the pathogen as *Elizabethkingia anophelis*, a closely related species to *E. meningoseptica*. Due to the unusual nature of the pathogen, an investigation of the patient’s eye drops, sinks and cubicle in the ward where the patient stayed was conducted for the presence of the pathogen. However, culture results did not indicate the presence of *E. anophelis* ruling out the possibility of nosocomial acquisition. This raised the possibility that alternate modes have been employed in the transmission of the bacterium leading to postoperative endophthalmitis. Our findings suggest that *E. anophelis* is a slow growing bacterium compared to *E. coli* and is capable of natural transformation during a narrow window of exponential phase of growth.

CG analysis with 25 other sequenced strains of *E. anophelis* showed several differences. Pan and core genome analysis revealed that the strain *E. anophelis* endophthalmitis has undergone massive gene rearrangements indicated by the high number of unique and exclusively absent genes predicted in its genome. Exclusion of *E. anophelis* endophthalmitis genome from the pan-core genome analysis has led to an increase in the core genome size by 37% indicating the strain’s divergence. The presence of a large number of MGEs and the horizontally acquired genes would have contributed to the genome rearrangement in the organism, diverging it from other analyzed genomes. These data along with others described below suggest the possibility of the recent emergence of the strain.

Survival in diverse environments such as mosquito mid gut and human tissues necessitates the bacterium to adapt to the respective niche environments. In such a scenario, possession of MGEs would help adjust and thrive. Analysis for MGEs revealed the presence of GIs, remnants of phage genomes and ICEs amongst several of the study genomes, indicating that the organisms with incomplete prophages and ICEs have further undergone gene gain/loss which may be of an evolutionary requirement for the pathogen to be successful in diverse ecological niches. Interplay between the bacterial defence systems (RMs, CRISPRs) and anti-RM proteins are known to decide the fate of the cell towards or against HGT^30^ (Supplementary Table 12). Taken together, these observations highlight the potential of the organism to be involved in more robust HGT. The presence of T6SS^iii^ and T4SS secretome further confirms that HGT is a norm and not an exception in *E. anophelis*. Given this scenario, it is not surprising to find several genes associated with antibiotic resistance, efflux pumps, virulence factors and competence. The finding of an exclusive virulence gene, arylsulfatase in the organism along with other genes with potential for pathogenesis and disease in humans, may provide further insights into the adaptation mechanisms of the pathogen to thrive under diverse ecological niches.

Notably, the virulence factor arylsulfatase with potential to cause meningitis was not found in any of the other analysed genomes including those from the central african republic outbreak (Po0527107 and V0378064) associated with neonatal meningitis^8^. This indicates that the current study strain may also have the potential to cause meningitis and there may be other meningital virulence factors that are yet to be identified and characterized.

From the features described, it is evident that *E. anophelis* endophthalmitis is a novel human pathogen with distinct characteristics. It is tempting to speculate how the organism could have been transmitted possibly by the mosquito vector to cause eye infection hitherto not associated with *E. anophelis*. Different mosquito species are highly prevalent in India, Africa and elsewhere. Thus, it is not unrealistic to suggest the transmission of the pathogen to human hosts by the mosquito vector in whose gut *E. anophelis* was first discovered. We speculate that the mosquito may have bitten near the eye after the patient has undergone surgery, thus transmitting the bacterium into the host’s ocular system. However, there could be alternate explanations which could account for the transmission. Also, further investigations are needed to confirm the transmission routes including zoonotic transmission of this enigmatic pathogen.

Given the enhanced antimicrobial resistance observed in *E. anophelis*, and its capacity to thrive in many ecological habitats it is very critical to implement best healthcare practices when in contact with the pathogen and initiate appropriate surveillance measures before the pathogen gets involved in the next outbreak.

## Methods

### Case presentation and strain characterization

A 67-year-old patient with complaints of redness, pain and loss of vision in the right eye after cataract surgery was admitted to the Department of Ophthalmology, Sri Sathya Sai Institute of Higher Medical Sciences, Prasanthigram, India. *E. anophelis* was isolated from the vitreous fluid of the infected eye^12^. Identification, antibiotic susceptibility testing (AST N281 card) and determination of Minimum Inhibitory Concentration (MIC’s) were performed using Vitek 2 (BioMérieux, France) as per CLSI (Clinical and Laboratory Standards Institute) guidelines.

### Natural transformation

*E. anophelis* endophthalmitis cultures were harvested at an OD600 of 0.37, 0.54, 0.61, 0.75 and 0.84. A plasmid (pCMV6-BDNF) containing eukaryotic Brain Derived Neurotrophic Factor (BDNF) with an ampicillin selection marker was added at 0 ng/mL, 28.2 ng/mL, 56.4 ng/mL and 84.6 ng/mL concentrations and incubated for 90 minutes at 30 °C. Untransformed *E. anophelis* endophthalmitis was found to exhibit resistance to ampicillin (250 µg/mL). Hence, transformation mixtures were plated on LB-amp agar plates at a higher concentration (600 µg/mL) in three dilutions, i.e., 1:2, 1:20 and 1:100. PCR was performed for the presence of BDNF gene in the plasmids isolated from the transformaned colonies using specific primers (forward Primer: 5′-GGATCCATGACCATCCTTTTCCTTACTATGG-3′; reverse Primer: 5′-AAGCTTCTATCTTCCCCTTTTAATGGTCAGT-3′) in a final volume of 20 μl containing 10 μl of PCR Master Mix (Takara) which includes dNTPs, MgCl2, *Taq* DNA polymerase and PCR buffer), 0.5 μM of forward and reverse primers, template DNA (2 μl) and nuclease free Water (4 μl). The PCR conditions employed for the amplification of BDNF gene were 94 °C - 2 minutes followed by 30 cycles of 94 °C - 15 seconds, 55 °C - 30 seconds, 72 °C - 47 seconds and a final elongation at 72 °C - 7 minutes. PCR amplicons were analysed on 1.2% agarose gel. *E.coli* DH5α and untransformed *E. anophelis endophthalmitis* served as negative controls.

### Growth kinetics

To determine the growth curve of *E. anophelis* endophthalmitis, 1% inoculum from overnight culture was added to 100 ml of fresh LB medium and incubated at 37 °C. Absorbance at 600 nm was measured at every 30 minutes. Growth kinetics revealed that the generation time of the isolate is 78 minutes at 37 °C (Supplementary Fig. 2).

### Genome characterization

Draft genome sequencing and assembly of *E. anophelis endophthalmitis* genome was recently reported^12^. The contigs from the draft assembly were subjected to gene prediction using PRODIGAL tool^31^ with default parameters as recommended. Predicted protein sequences were annotated using BLASTp against UNIPROT bacterial proteins database with evalue < = 0.001, > = 70% as query coverage and %Identity > = 30. *E. anophelis* NUHP1 (gi Number: 675102482) genome was used to construct the genome map of *E. anophelis endophthalmitis*. This led to successful organization of 111 contigs out of 167 contigs. A total of 3,729 ORFs encoding 2302 proteins were predicted from the assembled genome. All available (twenty-five other) *E. anophelis* genome sequences (as on February 02, 2017) were obtained from NCBI Genomes database for CG analysis and RAST (version 2.0) annotation has been repeated to obtain unambiguous results (Supplementary Table 1).

Bacterial Pan Genome Analysis (BPGA)^32^ was used for comprehensive pan/core genome analysis, functional annotation of the core, accessory and unique genes to Cluster of Orthologous groups (COG) categories and Kyoto Encyclopedia of Genes and Genomes (KEGG) pathways using default parameters. Phage genomes were identified by PHASTER^33^. Antibiotic resistance genes were predicted by RAST (version 2.0)^34^, Resfinder (version 3.0)^35^ and VRprofile (version 2.0)^36^. Virulence factors, genomic islands, Insertion sequences and T6SS Secretory systems were analyzed by VRprofile (version 2.0). T4SS genes were identified by SecReT4 (version 1.0)^37^. ICEberg (version 1.0)^38^ was used to screen for ICEs. Restriction Modification (RM) and anti-restriction systems were identified by RAST (version 2.0). CRISPRfinder^39^ was used to predict potential CRISPR gene clusters. Unless otherwise mentioned, all the above mentioned analyses were performed using default parameters.

## Electronic supplementary material

Supplementary information

List of 26 E. anophelis genomes used for comparative genomic analysis after RAST annotation

Genes associated with resistance to antibiotics and toxic compounds

Genes associated with putative virulence factors and anti-virulence

Putative prophage regions in the 26 E. anophelis genomes

Genomic islands that have been predicted in the study genomes

T6SS components that have been predicted in the study genomes

T4SS components that have been predicted among the study genomes

Predicted Restriction-Modification systems (RMs) in the 26 E. anophelis genomes.

Putative CRISPRs found in the 26 E. anophelis genomes

Predicted Anti-restriction proteins in the 26 E. anophelis genomes

Putative competence related genes

Overview of the Bacterial defence systems (Restriction-Modification systems, CRISPRs) and Anti-RM proteins in the study genomes

## References

[1] King EO (1959). Studies on a group of previously unclassified bacteria associated with meningitis in infants. American journal of clinical pathology.

[2] Jean SS, Lee WS, Chen FL, Ou TY, Hsueh PR (2014). Elizabethkingia meningoseptica: an important emerging pathogen causing healthcare-associated infections. J Hosp Infect.

[3] Pereira GH, Garcia Dde O, Abboud CS, Barbosa VL, Silva PS (2013). Nosocomial infections caused by Elizabethkingia meningoseptica: an emergent pathogen. Braz J Infect Dis.

[4] Li Y (2003). Chryseobacterium miricola sp. nov., a novel species isolated from condensation water of space station Mir. Systematic and applied microbiology.

[5] Green O, Murray P, Gea-Banacloche JC (2008). Sepsis caused by Elizabethkingia miricola successfully treated with tigecycline and levofloxacin. Diagnostic microbiology and infectious disease.

[6] Kampfer P, Busse HJ, McInroy JA, Glaeser SP (2015). Elizabethkingia endophytica sp. nov., isolated from Zea mays and emended description of Elizabethkingia anophelis. International journal of systematic and evolutionary microbiology.

[7] Kampfer P (2011). Elizabethkingia anophelis sp. nov., isolated from the midgut of the mosquito Anopheles gambiae. International journal of systematic and evolutionary microbiology.

[8] Frank T (2013). First case of Elizabethkingia anophelis meningitis in the Central African Republic. Lancet.

[9] Teo J (2013). First case of E anophelis outbreak in an intensive-care unit. The Lancet.

[10] Lau SK (2016). Elizabethkingia anophelis bacteremia is associated with clinically significant infections and high mortality. Sci Rep.

[11] Chen GX, Zhang R, Zhou HW (2006). Heterogeneity of metallo-beta-lactamases in clinical isolates of Chryseobacterium meningosepticum from Hangzhou, China. J Antimicrob Chemother.

[12] Pradeep BE (2016). Correction for Pradeep *et al*., Draft Genome Sequence of Elizabethkingia anopheles, Isolated from a Postoperative Endophthalmitis Patient. Genome announcements.

[13] Perrin, A. *et al*. Evolutionary dynamics and genomic features of the Elizabethkingia anophelis 2015 to 2016 Wisconsin outbreak strain. **8**, 15483, 10.1038/ncomms15483 (2017). 10.1038/ncomms15483 PMC5458099 28537263

[14] Marchesini MI (2011). Brucella abortus Choloylglycine Hydrolase Affects Cell Envelope Composition and Host Cell Internalization. PloS one.

[15] Rensing C, Pribyl T, Nies DH (1997). New functions for the three subunits of the CzcCBA cation-proton antiporter. Journal of bacteriology.

[16] Du D, van Veen HW, Murakami S, Pos KM, Luisi BF (2015). Structure, mechanism and cooperation of bacterial multidrug transporters. Current Opinion in Structural Biology.

[17] Lau SK (2015). Evidence for Elizabethkingia anophelis transmission from mother to infant, Hong Kong. Emerging infectious diseases.

[18] Dong J (2013). Oroxylin A Inhibits Hemolysis via Hindering the Self-Assembly of α-Hemolysin Heptameric Transmembrane Pore. PLOS Computational Biology.

[19] Callegan MC, Jett BD, Hancock LE, Gilmore MS (1999). Role of Hemolysin BL in the Pathogenesis of Extraintestinal Bacillus cereus Infection Assessed in an Endophthalmitis Model. Infection and immunity.

[20] Beecher DJ, Pulido JS, Barney NP, Wong AC (1995). Extracellular virulence factors in Bacillus cereus endophthalmitis: methods and implication of involvement of hemolysin BL. Infection and immunity.

[21] Hume EBH, Dajcs JJ, Moreau JM, O’Callaghan RJ (2000). Immunization with Alpha-Toxin Toxoid Protects the Cornea against Tissue Damage during Experimental Staphylococcus aureus Keratitis. Infection and immunity.

[22] Hoffman JA, Badger JL, Zhang Y, Huang SH, Kim KS (2000). Escherichia coli K1 aslA contributes to invasion of brain microvascular endothelial cells *in vitro* and *in vivo*. Infect Immun.

[23] Bliven KA, Maurelli AT (2012). Antivirulence Genes: Insights into Pathogen Evolution through Gene Loss. Infection and Immunity.

[24] Records AR (2011). The Type VI Secretion System: A Multipurpose Delivery System with a Phage-Like Machinery. Molecular Plant-Microbe Interactions.

[25] Russell AB (2014). A type VI secretion-related pathway in Bacteroidetes mediates interbacterial antagonism. Cell host & microbe.

[26] Breurec S (2016). Genomic epidemiology and global diversity of the emerging bacterial pathogen Elizabethkingia anophelis. Scientific Reports.

[27] Vasu K, Nagaraja V (2013). Diverse Functions of Restriction-Modification Systems in Addition to Cellular Defense. Microbiology and Molecular Biology Reviews: MMBR.

[28] Bickle TA, Krüger DH (1993). Biology of DNA restriction. Microbiological Reviews.

[29] McMahon SA (2009). Extensive DNA mimicry by the ArdA anti-restriction protein and its role in the spread of antibiotic resistance. Nucleic acids research.

[30] Stern A, Sorek R (2011). The phage-host arms-race: Shaping the evolution of microbes. Bioessays.

[31] Hyatt D (2010). Prodigal: prokaryotic gene recognition and translation initiation site identification. BMC bioinformatics.

[32] Chaudhari NM, Gupta VK, Dutta C (2016). BPGA- an ultra-fast pan-genome analysis pipeline. Scientific Reports.

[33] Arndt D (2016). PHASTER: a better, faster version of the PHAST phage search tool. Nucleic Acids Research.

[34] Aziz RK (2008). The RAST Server: Rapid Annotations using Subsystems Technology. BMC Genomics.

[35] Zankari E (2012). Identification of acquired antimicrobial resistance genes. Journal of Antimicrobial Chemotherapy.

[36] Li, J. *et al*. VRprofile: gene-cluster-detection-based profiling of virulence and antibiotic resistance traits encoded within genome sequences of pathogenic bacteria. *Briefings in Bioinformatics*, 1–9, 10.1093/bib/bbw141 (2017). 10.1093/bib/bbw141 28077405

[37] Bi D (2013). SecReT4: a web-based bacterial type IV secretion system resource. Nucleic Acids Research.

[38] Bi D (2012). ICEberg: a web-based resource for integrative and conjugative elements found in Bacteria. Nucleic Acids Research.

[39] Grissa I, Vergnaud G, Pourcel C (2007). CRISPRFinder: a web tool to identify clustered regularly interspaced short palindromic repeats. Nucleic Acids Research.

