# Supplementary material for: Comparative genomic analysis of a naturally competent Elizabethkingia anophelis isolated from an eye infection
Source: Sci Rep. 2018 May 31;8:8447. doi: 10.1038/s41598-018-26874-8 (PMC5981450; doi:10.1038/s41598-018-26874-8)
Supplement: Supplementary file 1 — Supplementary information [file 41598_2018_26874_MOESM1_ESM.pdf]

Title:

Comparative genomic analysis of a naturally competent *Elizabethkingia anophelis* isolated from an eye infection

Bulagonda Eswarappa Pradeep[1*], Bhavani Manivannan[1], Niranjana Mahalingam[1,2], Manmath Lama[1], Pachi Pulusu Chanakya[1], Balaram Khamari[1], Sudhir Jadhao[3], Madavan Vasudevan[3] and Valakunja Nagaraja[4,5*]

1. Department of Biosciences, Sri Sathya Sai Institute of Higher Learning, Prasanthi Nilayam, Puttaparthi, Andhra Pradesh, India.
2. Department of Microbiology, Sri Sathya Sai Institute of Higher Medical Sciences, Prasanthigram, Andhra Pradesh, India.
3. Bionivid technology Pvt Ltd, 4C-209, 4th cross, Kasturi Nagar, Bengaluru, India.
4. Department of Microbiology and Cell Biology, Indian Institute of Science, Bengaluru, India.
5. Jawaharlal Nehru Centre for Advanced Scientific Research, Bengaluru, India.

Running title: *Elizabethkingia anophelis* genomic analysis

Corresponding authors*:

*Bulagonda Eswarappa Pradeep, Department of Biosciences, Sri Sathya Sai Institute of Higher Learning, Prasanthi Nilayam, India. 515134.

Tel no: +91-8008739138; Fax: +91-8555 286919. Email: bepradeep@sssihl.edu.in

*Valakunja Nagaraja, Department of Microbiology and Cell Biology, Indian Institute of Science, Bengaluru, 560012 and Jawaharlal Nehru Centre for Advanced Scientific Research, Bengaluru. 560064.

Email: vraj@iisc.ac.in

Keywords –*Elizabethkingia anophelis*, Endophthalmitis, Comparative genomics (CG), Horizontal Gene Transfer (HGT), Mobile Genetic Elements (MGEs).

Supplementary Table 1: List of 26 *E. anophelis* genomes used for comparative genomic analysis after RAST annotation.

Supplementary Table 2: Genes associated with resistance to antibiotics and toxic compounds

Supplementary Table 3: Genes associated with putative virulence factors and anti-virulence

Supplementary Table 4: Putative prophage regions in the 26 *E. anophelis* genomes.

Prediction of the prophage regions was performed using the default parameters of the tool PHASTER. A total of 59 putative prophage regions have been predicted among the study isolates. Att- Attachment site.

Supplementary Table 5: Genomic islands that have been predicted in the study genomes.

Prediction of the genomic islands (GIs) was performed using the default parameters of the tool VRprofile. A total of 107 genomic islands have been predicted. Several antibiotic resistance genes (AR), Virulence factors (VF), Pathogenicity islands (PAI), Insertion sequences (IS), and Secretory systems (Sec Sys) have been identified inside the GIs.

Supplementary Table 6: T6SS components that have been predicted in the study genomes

Supplementary Table 7: T4SS components that have been predicted among the study genomes

Supplementary Table 8: Predicted Restriction-Modification systems (RMs) in the 26 *E. anophelis* genomes.

Supplementary Table 9: Putative CRISPRs found in the 26 *E. anophelis* genomes.

Supplementary Table 10: Predicted Anti-restriction proteins in the 26 *E. anophelis* genomes.

Supplementary Table 11: Putative competence related genes

Supplementary Table 12: Overview of the Bacterial defence systems (Restriction-Modification systems, CRISPRs) and Anti-RM proteins in the study genomes.

Supplementary Figure 1: Comparative analysis of *mutY* (Adenine DNA glycosylase) across the 26 study genomes.

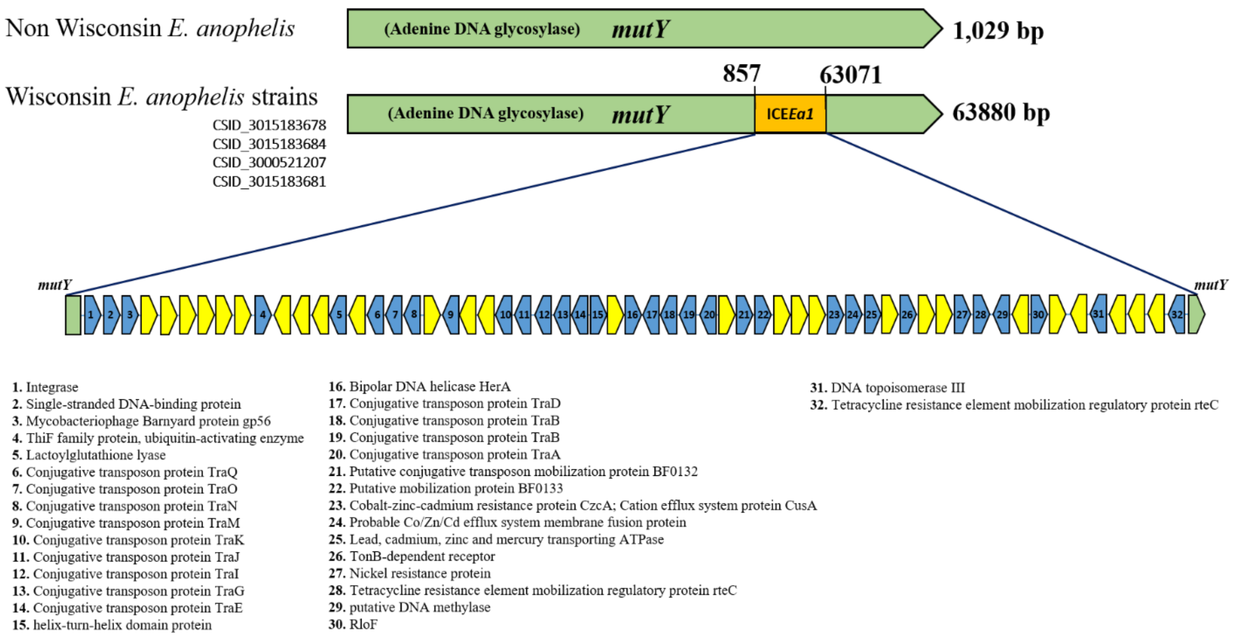

The gene *mutY* (Adenine DNA glycosylase) was found disrupted by a 62,214 bp long ICE in the four Wisconsin *E. anophelis* genomes. There was no disruption of *mutY* in the remaining twenty-two genomes. The gene cassette of the ICE in the disrupted *mutY* included 32 genes which are part of conjugative transposon, heavy metal and tetracycline resistances.

Supplementary Figure 2: Growth curve of *E. anophelis* endophthalmitis.

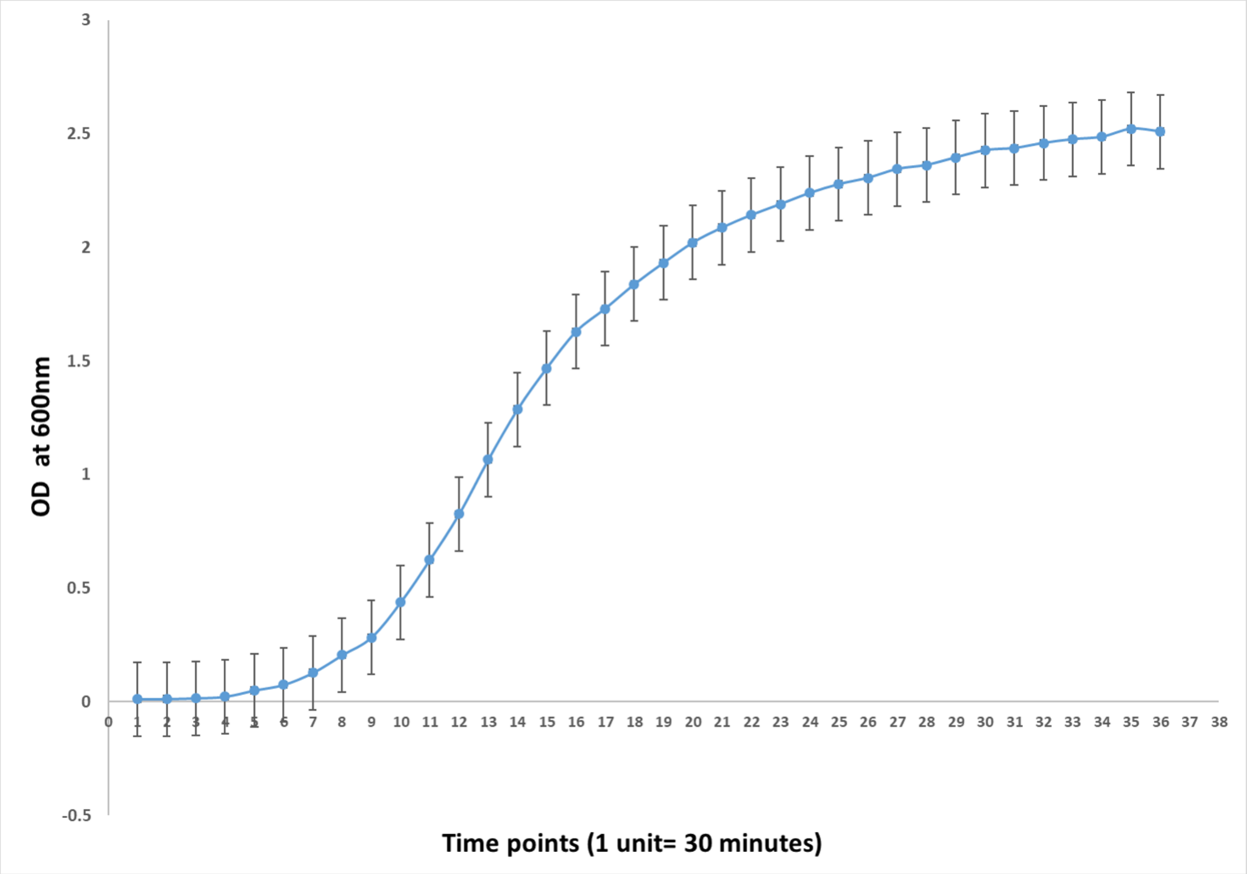

Growth was determined by measuring OD_{600} against the blank (LB medium). The initial concentration of the tested strains was 0.01175 at OD_{600}. Aliquots from five replicates were taken at intervals of 30 min after incubation at 37°C. Generation time has been calculated as the time taken to double the OD (as a measure of number of bacteria) during mid-log phase. For each time point, five independent growth curves were monitored and the data was fitted.
